# Maintaining Independence in People with Dementia who have had a fall: a pilot cluster randomised controlled trial: the Maintain study

**DOI:** 10.1002/alz70858_098951

**Published:** 2025-12-25

**Authors:** Louise M Allan, Leanne Greene, Bethany Whale, James Connors, Ashima Sharma, Alison Bingham, Abigail Hall, Claire M Hulme, Vicki M Goodwin, Sarah Morgan‐Trimmer

**Affiliations:** ^1^ University of Exeter, Exeter, Devon, United Kingdom; ^2^ University of Exeter, Exeter, United Kingdom

## Abstract

**Background:**

Falls are very common in dementia and lead to a loss of independence. Previous trials of interventions for falls in dementia have not aimed to maintain independence. This pilot cluster randomised controlled trial (RCT) examined the feasibility of a home‐based rehabilitation intervention designed to enhance independence in people with dementia who had fallen, aiming to inform the design of a future definitive RCT.

**Method:**

This study used a mixed‐methods design with an embedded process evaluation (reported separately) to assess the intervention's feasibility, fidelity, and acceptability. Participants over 50, diagnosed with dementia and a history of falls within the last 6 months, were recruited from six sites across the UK (randomised 1:1 by site to intervention or usual care). The intervention consisted of personalised rehabilitation sessions delivered by healthcare professionals, based on personal goals. Feasibility outcomes included recruitment, retention, adherence rates, and participant acceptability, with data collected through standardised measures.

**Result:**

Thirty‐one participants, with a mean age of 78 years (SD = 9.03), were recruited, (29 White British, 1 mixed race, 1 other). Of 54 individuals screened, 36 (66%) met eligibility criteria; among these, 36 were approached, and 31 (86%) of these consented to participate. 18 started the intervention and 13 started usual care. 27 underwent 3 month follow up and 25 underwent 6 month follow up. Among the intervention participants the mean initial assessment time was 2.05 hours, mean number of sessions was 15, range (6‐25). Primary outcome data collection was 96.8% at baseline and 100% at the 6‐month follow‐up.

**Conclusion:**

The Maintain intervention showed feasibility among people with dementia who had fallen, as evidenced by the trial processes for participants who entered the study. The progression criteria for going on to a definitive RCT to demonstrate the effectiveness and cost‐effectiveness of the intervention were met.

